# Combination of chick embryo and nutrient mixture prevent D-galactose-induced cognitive deficits, immune impairment and oxidative stress in aging rat model

**DOI:** 10.1038/s41598-019-40953-4

**Published:** 2019-03-11

**Authors:** Jia Ma, Huaxin Wang, Bing Liu, Yujia Shan, Huimin Zhou, Xia Qi, Wenguo Wu, Li Jia

**Affiliations:** 1 College of Laboratory Medicine, Dalian Medical University, Dalian, 116044 Liaoning Province China; 2 Department of Pathology and Forensic Medicine, Dalian Medical University, Dalian, 116044 Liaoning Province China; 3 Dalian Jinfu Biological Technology Development Co., Ltd, Dalian, 116000 Liaoning Province China

**Keywords:** Ageing, Ageing, Nutrition, Nutrition

## Abstract

Aging is spontaneous and inevitable processes that lead to changes in biological systems. The present paper was designed to investigate the anti-aging roles of chick embryo (CE) and nutrient mixture (NM) in aging rats. Aging was induced by administration of D-galactose (D-gal, 500 mg/kg/day for 90 days). CE and NM were administered to aging rats through different dose gavage once a day. Cognitive function assessment was performed using the Morris water maze test. At the end of experiment, serum and tissues were collected for immunity and antioxidation function. The organs and tissues were excised for histological study. The results demonstrated that CE plus NM was superior treatment to improve the histopathologic changes and reverse learning and memory impairment of the aging rats. CE plus NM also increased the spleen and thymus index as well as splenocyte proliferation, and reversed inflammatory cytokine levels. In addition, the biochemical index showed that CE plus NM could improve the antioxidant enzyme activity of the aging rats, decrease lipofuscin (LF) and glutamate content. CE plus NM also inhibited the activation of TLR4/NF-κB pathway stimulated by LPS in splenic B lymphocytes. Overall, these results seem to be implying that CE plus NM was used as potentially natural supplement or functional food for preventing aging.

## Introduction

Aging, as a natural phenomenon, leads to progressive deterioration of tissues and organs^1^. Anti-aging has already become a major public issue with the increasing elderly population in the world. Over the past decades, it is well established that the aging process is associated with antioxidant defense system dysfunction, immune system impairment, cognitive and motor degeneration^2^. Yet, the imbalance of free radical metabolism damages cellular lipids, proteins and DNA, which cause the body aging^3^. During the aging process, various pro-inflammatory molecules are generated to enhance inflammation cascade associated with different age-related pathologies^4^. In addition, antioxidant and immunostimulant have been reported to be beneficial to aging process^5^.

D-gal as a sugar, which completely metabolizes at normal concentration, is a natural agent in the body^6^. An excess of D-gal converts to aldose and hydrogen peroxide during the catalysis of galactose oxidase, and culminates in the generation of free radical, which eventually impairs cellular function^7^. It has been shown that D-gal induced animals express aging-associated changes in body physiology and phenotypes, such as cognitive dysfunction, increased oxidative stress, decreased antioxidant enzyme activity, diminished immune response and mitochondrial dysfunction^8^. Hence, D-galactose-induced aging model has been admitted by researchers and been widely used in studying aging mechanism^9^.

In recent years, numerous functional food have been found to possess potent anti-aging activities, and as potential candidate for the development of anti-aging therapies^10^. Nutrient mixture is widely used in China. The main component is amino acids, nucleotide, vitamin, trace elements, inositol, folate, etc. They have been shown for its biological activities, including anti-fatigue, antioxidant activity and improving immunity^11^. In addition, as natural dietary supplement, chick embryo eggs are considered as the nutrition, have quality supplement and illness treatment in China^12^. Studies have shown that chick embryo contains stem cell factor (SCF), nerve growth factor (NGF), epidermal growth factor (EGF), interleukin-4 (IL-4) and interleukin-2 (IL-2)^13,14^. These five factors have physiological activities, including maintaining neuron survival, activating the immune system and repairing tissue damage. In particular, they contribute to antioxidant capacity for the aged^15–17^. So, it is interesting to investigate whether the CE and NM possess any anti-aging effect *in vitro* and *in vivo*.

In this study, the aging rat model induced by D-gal was used to evaluate the effect of CE and NM against aging *in vitro* and *in vivo*. CE and NM were administered to aging model rats through different dose. The related-function of aging rats was investigated. It was well confirmed that CE plus NM may contribute to the improvement of aging and serve as a recuperative support care for aging process in rats.

## Results

### The levels of SCF, NGF, EGF, IL-2 and IL-4 in chick embryo eggs

**Figure 1:**
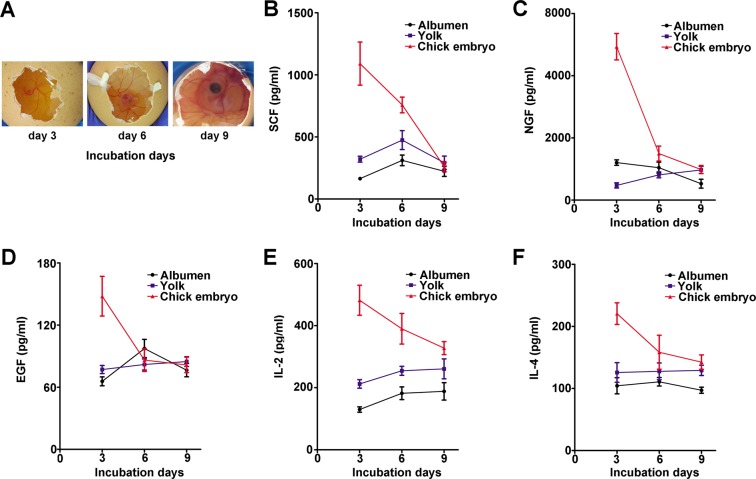
Chick embryo growth was observed at 3 days, 6 days and 9 days (**A**). The change of growth factors levels in the albumen, yolk and chick embryo of eggs during incubation. (**B–F**) The levels of SCF, NGF, EGF, IL-2 and IL-4 were examined by using ELISA kit. These cytokines were particularly rich in chick embryo at 3 days compared with the other groups (P < 0.05). Data are presented as mean ± SD of three independent experiments.

The levels of SCF, NGF, EGF, IL-2 and IL-4 were analyzed in Fig. 1. As shown in Fig. 1A, the growth dynamics of chick embryo was observed at 3 days, 6 days and 9 days. The level of SCF differed among chick embryo, yolk and albumen. The SCF level was the highest in the chick embryo on the third day of incubation (Fig. 1B). A higher NGF level was detected on the third day (Fig. 1C). NGF level was higher in chick embryo than those in yolk and albumen on the third day. Figure 1D showed EGF level was the highest on the third day. Further analysis revealed that the EGF level was different among the chick embryo, yolk and albumen, and a high amount of EGF was available in the chick embryo. IL-2 (Fig. 1E) and IL-4 (Fig. 1F) in chick embryo had the highest levels at 3 days. In brief, we found that SCF, NGF, EGF, IL-2 and IL-4 levels from chick embryo were particularly rich at 3 days.

### CE and NM affect the histopathology in aging rats

**Figure 2:**
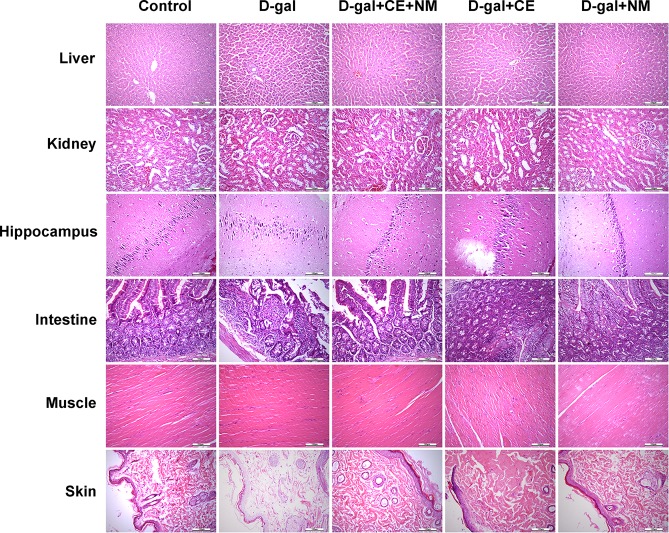
The effect of CE and NM supplement on histopathological changes of aging rats. The liver, kidney, brain, intestine, muscle and skin were removed from rats of each group after CE and NM treatment. Sections were processed for HE staining. Representative histopathological image at high magnification are shown (black bar, 100 μm).

To confirm whether CE and NM have a protective effect on aging rats, the liver, kidney, brain, intestine, muscle and skin tissues were detected. As shown in Fig. 2, liver tissue from control group showed normal structure of hepatic lobules with a central vein, radiating cords of hepatocytes and prominent round nuclei. Compared to the control group, histopathological examination of liver from D-gal induced rats showed an obvious injury. The hepatic lobules structure was not clear. Hepatic sinusoids expanded, hepatic cord arranged mussily and a lot of lipofuscins deposited. The hepatocytes presented several types of pathological damage such as cytoplasmic rarities, karyopyknosis and chromatinic anachromasis. However, the structure of hepatic lobules improved obviously, hepatocytes cord-like permutation was orderly and a little lipofuscins deposited in CE and NM administration groups. The improvement was more significant in the CE combined with NM group.

Histopathological changes in kidney sections were examined (Fig. 2). Kidney sections from D-gal group showed tubular cell damage and several glomeruli atrophic changes. In comparison with the D-gal group, tubular cell damage and glomerular changes in the kidney were improved in the intake of CE and NM groups. However, CE plus NM could better improve kidney damage.

The hippocampus of control group showed no remarkable abnormalities. Hippocampal neuron cells were arranged neatly with no noticeable cell loss (Fig. 2). By contrast, atrophy and loss of neurons in the hippocampus were observed in the D-gal group. These changes were substantially attenuated in the intake of CE and NM groups. However, the improved effect of CE plus NM on hippocampus was more notable.

Intestine tissue samples were stained with HE and shown in Fig. 2. Compared with the control group, the D-gal group exhibited sparse intestinal villi and structural damage, as well as the muscular layer were very thin. The abnormal intestinal villus and muscular layer structure was improved in the intake of CE and NM groups, while the combination of CE and NM could better ameliorate intestine damage.

Muscle sections from the control group showed normal muscle fiber structure. Following CE and NM administration, obvious reduction of the signs of injury was observed, especially in CE plus NM treatment group, the close arrangement of muscle fibers was similar to those in the control group (Fig. 2).

D-gal group showed significant changes in skin samples compared with control group by HE staining (Fig. 2). The rats of D-gal group showed that the dermal layer was loose, collagen matrix structure changed and fibroblasts reduced. Moreover, change was attenuated by long-term treatment of CE and NM. The number of fibroblast and collagen level were increased, whereas, the CE plus NM treatment could better reverse the changes. In addition, sections of the spleen, stomach, pancreas, and heart showed no structural abnormities in all groups (data not shown). These results showed that CE plus NM could better protect D-gal induced liver, kidney, brain, intestine, muscle, skin damages in aging rats.

The content of MDA and the activity of SOD were detected in liver, kidney and hippocampus tissues. Compared with the control group, the content of MDA was increased and the activity of SOD was decreased in the D-gal group (Fig. S1, ^#^P < 0.05). However, Exposure to CE and NM significantly decreased the content of MDA and increased the activity of SOD compared to D-gal group (*P < 0.05). These data indicated that combination of CE and NM could better improve the oxidative stress levels of liver, kidney and hippocampus tissues in aging rats.

### CE and NM affect the body weight and immune function in aging rats

**Figure 3:**
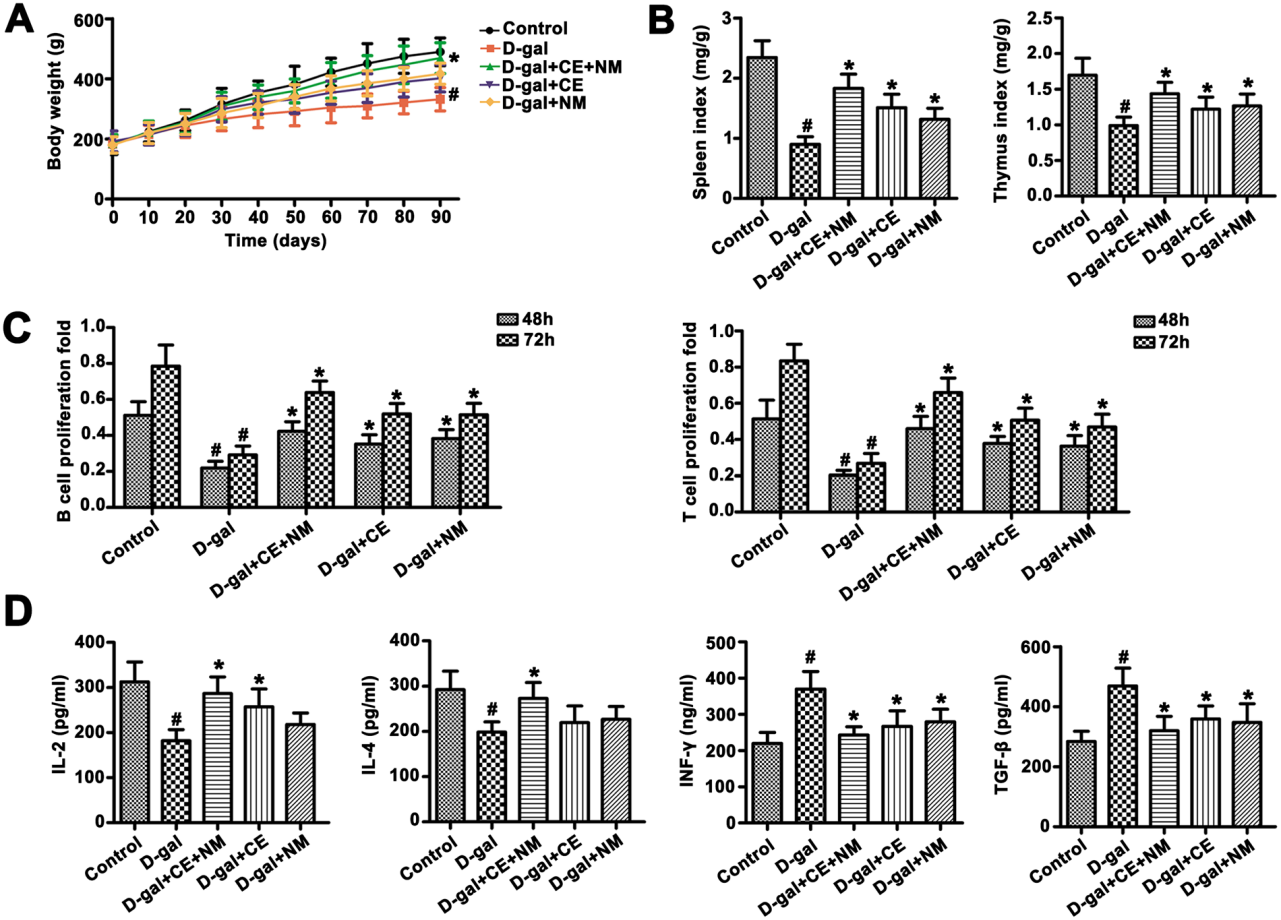
CE and NM supplement effect on body weight and immune function in aging rats. (**A**) The effects of CE and NM on the changes of body weight were measured during the 90 days study. (**B**) CE and NM treatment increased the indices spleen and thymus of immunosuppressed rats. (**C**) Spleen cells were seeded to 96-well plates with ConA (10 μg/mL) or LPS (10 μg/mL) and the viability was determined by CCK-8 assay at 48 and 72 h. CE and NM treatment stimulated B and T lymphocyte proliferation of splenocyte. (**D**) IL-2, IL-4, INF-γ and TGF-β serum concentrations were identified from different groups. ^#^P < 0.05 versus control groups. *P < 0.05 versus D-gal groups. Data are presented as mean ± SD.

CE and NM were administered to rats for 90 days, and its effects on the average body weight and immune function are presented in Fig. 3. Body weight of D-gal group was reduced compared to that of control group (^#^P < 0.05). Body weight of rats in the D-gal + CE + NM group was obviously higher than that in the D-gal group (*P < 0.05). Compared with D-gal group, the intake of CE or NM did not alter significantly body weight (Fig. 3A).

As important immune organs, the spleen and thymus weights are intuitive index in the immune function. The spleen and thymus index were decreased in the D-gal group (Fig. 3B). The spleen and thymus index was rebounded by CE and NM treatment, while CE plus NM was a superior treatment.

The induction of splenocyte proliferation by CE and NM treatment was shown in Fig. 3C. The D-gal suppressed the B and T lymphocyte proliferation of splenocyte at 48 or 72 h. The proliferative ability of B and T lymphocytes in CE + NM group was higher than that in the D-gal group. Furthermore, CE plus NM facilitated faster B and T lymphocyte proliferation of splenocyte.

The IL-2 and IL-4 levels were obviously lower in the D-gal group than that in the control group (Fig. 3D). The D-gal + CE + NM group showed a significant increase in the IL-2 and IL-4 levels compared to the D-gal group (^#^P < 0.05). The IL-2 level increased in the D-gal + CE group (*P < 0.05), the IL-4 level was a slight increase in the D-gal + CE group compared to the D-gal group. However, the IFN-γ and TGF-β levels in the D-gal group was higher than that in the control group (^#^P < 0.05). In contrast, both CE and NM administration suppressed IFN-γ and TGF-β levels (*P < 0.05). Notably, CE plus NM administration preferably reversed inflammatory cytokine levels in the serum.

### CE and NM affect the antioxidant enzyme activity and LF content in brain and liver tissues in aging rats

**Figure 4:**
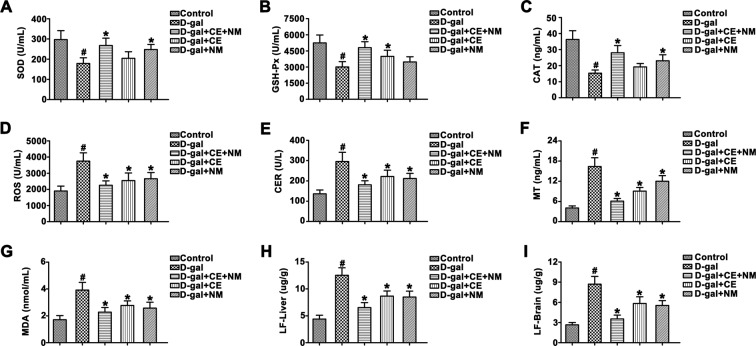
CE and NM affect the antioxidant index and LF level in aging rats. (**A**) The serum level of SOD was tested using ELISA. (**B**) The content of GSH-Px in serum was measured. (**C**) The serum level of CAT was examined. (**D**) ROS content in serum was determined. (**E**) Serum CER content was calculated. (**F**) The level of serum MT was detected. (**G**) MDA level in serum was calculated. (**H**) The change of LF content in liver was measured. (**I**) The content of LF in brain was detected. ^#^P < 0.05 versus control groups. *P < 0.05 versus D-gal groups. Data are expressed as mean ± SD of three independent experiments.

In the D-gal group, the activity of SOD, GSH-PX and CAT in the serum were lower than that in control group (Fig. 4A–C, ^#^P < 0.05), suggesting that the aging model was built as expected successfully. The activity of SOD and CAT in the D-gal + CE + NM group and D-gal + NM group was higher than that in the D-gal group (*P < 0.05), but the levels were slight increase in D-gal + CE group. The activity of GSH-PX in the D-gal + CE + NM group and D-gal + CE group was higher than that in the D-gal group (*P < 0.05).

In the D-gal group, the contents of ROS, CER, MT and MDA were higher than that in control group (Fig. 4D–G, ^#^P < 0.05). Interestingly, treatment of rats with CE and NM decreased the contents of ROS, CER, MT and MDA in the serum (*P < 0.05), while combination of CE and NM could better reduce these cytokine levels of serum in D-gal-induced rat.

LF content of brain and liver tissue were shown in Fig. 4H,I. LF content of brain and liver tissues were increased in the D-gal group, and those were decreased in the intake of CE and NM groups (*P < 0.05). The content of LF was notably reduced in D-gal + CE + NM group.

### CE and NM affect the Morris water maze test in aging rats

**Figure 5:**
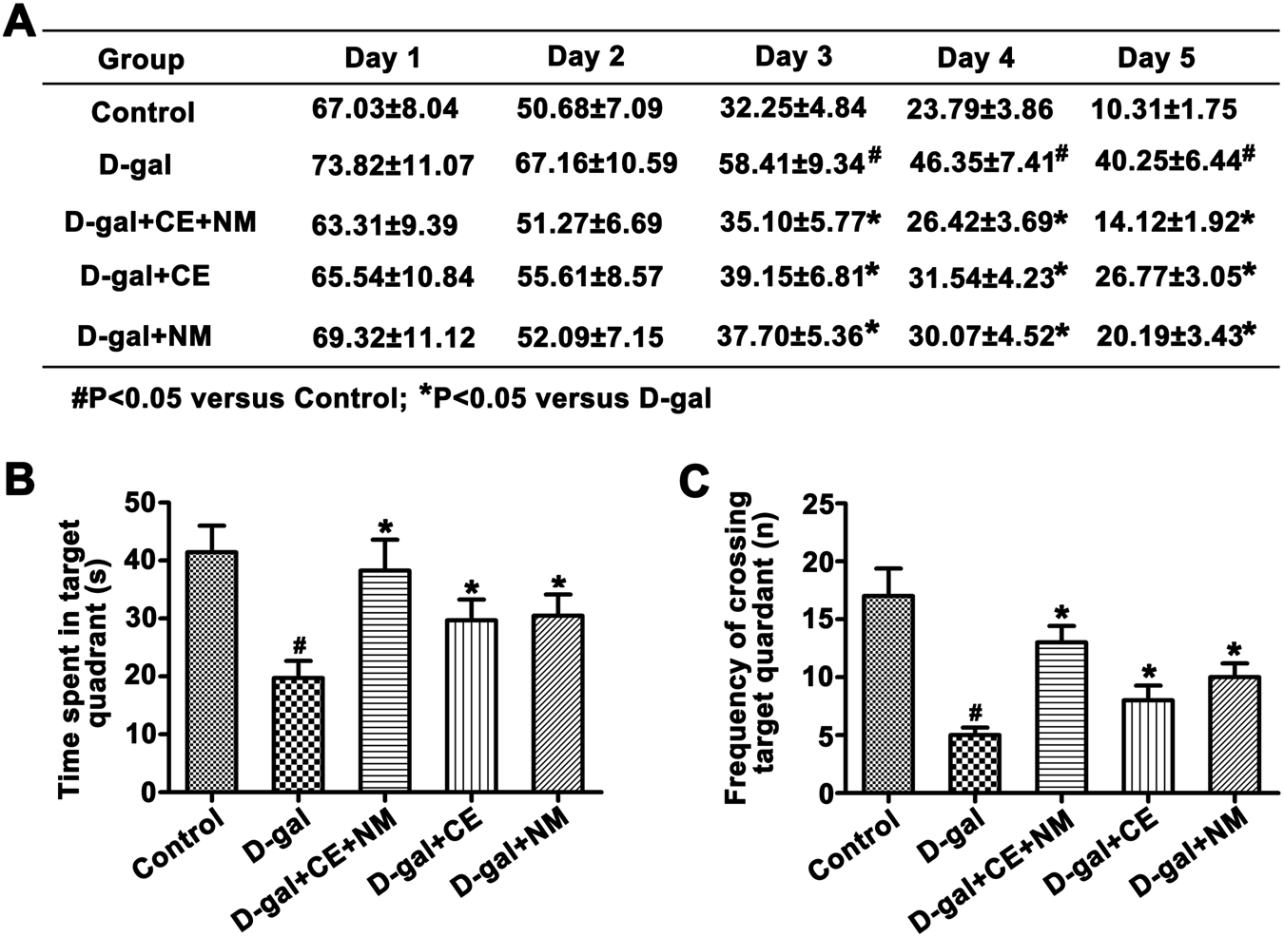
Spatial learning and memory in the Morris water-maze test. (**A**) Mean escape latency to platform was recorded during five training days in the hidden platform test. (**B**) The time spent in the quadrant where the platform was once placed. (**C**) The frequency of crossing the target quadrant in the probe trial. ^#^P < 0.05 versus control groups. *P < 0.05 versus D-gal groups. Values are expressed as mean ± SD for rats from each group.

Morris water maze test was performed to detect the effect of CE and NM on spatial learning and memory ability. The results showed that the escape latency of all groups gradually decreased in varying degree along with the increase of training day (Fig. 5A). Compared with the control group, the D-gal group took a longer time to find the hidden platform after the second day training (^#^P < 0.05). Meanwhile, the prolonged escape latency in the D-gal-induced rats was reduced by long-term administration of CE and NM (*P < 0.05). In the probe trial, the total time spent swimming in the target quadrant and the frequency crossing over the target quadrant were lower in the D-gal group than that in control group (Fig. 5B,C, ^#^P < 0.05). The intake of CE and NM increased the time spent in the target quadrant and frequency crossing over the target quadrant than in the D-gal group (*P < 0.05). However, CE plus NM treatment had a greater effect to reverse the deficit of spatial learning and memory.

### CE and NM affect the glutamate content in aging rats

Compared with the control group, glutamate content of brain was increased in D-gal group (Fig. S2, ^#^P < 0.05). However, glutamate content of brain was decreased in CE and NM administration groups than D-gal group (*P < 0.05). By contrast, glutamate content of brain was significantly reversed in D-gal + CE + NM group.

### CE and NM affect the activation of TLR4/NF-κB pathway stimulated by LPS in splenic B lymphocytes

As TLR4 pathway serves a key role in inflammatory response of immune cells. To further examine the function of the TLR4 signaling pathway in splenic B lymphocytes, rat splenic B lymphocytes were isolated and treated with LPS. As shown in Fig. S3, the expression of TLR4 and NF-κB p65 in LPS stimulated splenic B lymphocytes were significantly increased in D-gal group, compared to the control group. Moreover, a regulatory role of CE and NM treatment on TLR4 and NF-κB expression in rat splenic B lymphocytes was also observed. CE or NM administration reduced TLR4 and NF-κB p65 levels, while TLR4 and NF-κB p65 expression was also decreased dramatically in CE plus NM administration group, upon LPS treatment. These finding suggested that CE and NM administration could decrease LPS-induced activation of NF-κB pathway triggered by TLR4 in rat splenic B lymphocytes.

## Discussion

Chick embryo eggs have been recognized as a plentiful source of nutrients and contain different bioactive substance^18^. Previous data have indicated that chick embryo eggs had high antioxidant activity and prevent premature aging^19^. Scholars have confirmed that chick embryo eggs hydrolysates increased the spleen index, promoted proliferation of rats lymphocytic, enhanced hemolysin activity and macrophage phagocytic capacity, as well as prolonged exhaustive lethal swimming time^20^. Furthermore, Cherian G. *et al*. demonstrated the chick embryo relied on nutrients deposited by the hen in the egg for sustaining over one-third its life^21^. Nutrients within the embryo change with the incubation time. In the present study, on the third day of incubation, the growth factors isolated from chick embryo exhibited high levels. This might be explained by maternal nutrition and immunity provided primary protection to the developing embryo, which in turn progressively degraded the growth factors to form embryonic tissue^22^. In addition, Karim MR *et al*. demonstrated the supplement with vitamin C and E might provide protection against dementia and improve cognitive function in late life^23^. Soybean phospholipid had also potent anti-oxidant, anti-inflammatory and anti-aging activity, providing new insights into the application of resistant aging^24^. Furthermore, recent studies indicated that taurine was beneficial against a variety of aging-related diseases, such as chronic heart failure, diabetes, atherosclerosis, etc.^25^. Thus, we chose chick embryo from the third day of incubation and nutrients mixture (52 ingredients, including different amino acids, nucleotide, vitamin, folate, soybean phospholipid, inositol, iron, zinc, manganese, etc.) to evaluate the anti-aging effect.

D-gal is now recognized as an inducer of aging reagents that can be accelerated senescence in mice^26^. Generally, D-gal induced rat exhibit histopathological lesion, poor immune function, decreased in antioxidase activities and cognitive deficits^27^. The liver, kidney, brain, intestine, muscle and skin are important organs and tissues. However, their function gradually declined due to age-associated structural atrophy and impairment^28^. In this study, the improvement of organs and tissues damage was shown following CE and NM treatment, while the improvement of CE plus NM treatment was more remarkable. These data indicated that the concomitant of CE and NM might be the best way to exert their anti-aging action in aging model. SOD catalyzes the conversion of superoxide anion into hydrogen peroxide and oxygen, which is regarded as the first line of the antioxidant defense system^29^. MDA is an end-product of ROS-induced peroxidation, and it is another well-known indicator reflecting the levels of oxidative stress^30^. Previous studies have shown that indicate markedly aggravated oxidative stress and depletion of endogenous antioxidants in D-gal-induced rats^31^. However, treatment CE plus NM increased the activity of SOD and reduced the level of MDA in liver, kidney and hippocampus of D-gal induced rats. These results of biochemical analysis corresponded well with the histological assessments. It was reported that long-term administration of D-gal decreased the average weight of the animals^32^. We found that the average body weight of D-gal induced rats tended to be significantly increased after CE plus NM treatment, suggesting that CE plus NM affected body mass gain.

During aging, immune system is thoroughly participated in whole aging course of the organism^33^. Thymus and spleen, as important immune organ, are closely related to body aging^34^. The decreased thymus and spleen index indicated that cellular proliferation, differentiation and immune function were downregulated, which led to imbalance of cytokines in the body^35^. Correspondingly, the findings of the current study revealed CE plus NM could delay the atrophy of thymus and spleen of aging rats, together with enhanced splenocyte proliferation, thereby reduced the levels of INF-γ and TGF-β and increased the levels of IL-2, IL-4 in the D-gal induced rats, so as to delay immune function aging to a certain extent.

Free radical theory holds that because of an imbalance between free radicals and antioxidants, the structure and function of tissues and organs are in disorder, which leads to aging^36^. The antioxidant activity has been reported to be determined by the degree of oxidative stress^37^. In the current study, D-gal induced rats caused notable oxidative damage, including a decreased in SOD, GSH-Px and CAT levels, as well as increased in ROS, CER, MT and MDA levels in rat serum. These results were corresponding to previous reports^38^. Here, our result indicated that treatment with CE plus NM reversed the changes of SOD, GSH-Px, CAT, ROS, CER, MT and MDA levels. Moreover, related articles showed that age-related accumulation of damaged might promote oxidative stress dramatically, which could also enhance LF formation^39^. We also observed that accompanying with the decreased of ROS, CER, MT and MDA levels in the rat serum, and the level of LF was decreased. Therefore, CE plus NM might be restore the antioxidant defense system by increasing the activity of antioxidant enzymes, which led to low level of oxidative damage and mediated LF level.

It is reported that behavioral changes are more sensitive in the evaluation of cognitive and memory impairments, instead of neuro-toxicity related neuro-chemical alteration^40^. In this study, the spatial learning and memory ability of the D-gal induced rats was impaired, which was in agreement with previous findings^41^. The administration of CE plus NM reduced the escape latency, increased the time spent in the target quadrant and the frequency of crossing target quadrant, suggesting that CE plus NM had the potential to ameliorate cognitive deficits induced by D-gal. Glutamate is the critical excitatory neurotransmitter in the central nervous system and plays an important role in learning, memory, and cognition^42^. Excitotoxicity caused by glutamate, seem to be important factor involved in process of aging^43^. In this study, we found that CE plus NM supplement could decrease glutamate content. Therefore, CE plus NM treatment could serve a protective role in brain damage.

TLR4 engages all four toll-interleukin receptor (TIR) adaptors proteins to signing the chain reaction needed to activate intracellular signaling inflammatory response^44^. TLR4 is often expressed in inflammatory cells, which eventually activates NF-κB via cellular signaling cascades^45^. Our study found that CE and NM treatment effectively downregulated the expression of TLR4 NF-κB p65 in LPS stimulated splenic B lymphocytes. Consequently, the inhibitory role of CE and NM treatment on the inflammatory cytokines might partly pass through down-regulating the TLR4/NF-κB pathway.

In summary, our findings support the hypothesis that CE plus NM treatment plays an important role in improving cognitive degeneration, enhancing immune function and decreasing oxidative stress in aging rats. Based on these results, supplement with dietary CE plus NM may lead to the development of novel therapeutic approaches for anti-aging.

## Materials and Methods

### Chick embryo extract

Experiment was approved by the Animal Studies Ethics Committee of the Dalian Medical University, China. Chick embryo eggs were obtained from Dalian Jinfu Biological Technology Development Co., Ltd (Dalian, China). The eggs were incubated at 37.8 °C with the relative humidity of 60–65%. Chick embryo, yolk and albumen were sampled from eggs that were obtained on the third, sixth and ninth days. The SCF, NGF, EGF, IL-2 and IL-4 from extract were detected using enzyme-linked immunosorbent assay (ELISA, Lifespan Biosciences Co., Seattle, USA). The chick embryo was selected and frozen for further experiments.

### Nutrient mixture administration

NM was provided by Dalian Jinfu Biological Technology Development Co., Ltd (Dalian, China (Table 1). According to the proto-prescription, oral application was prepared.

**Table 1:** Composition, content, and proportion of nutrient mixture.

Sequence number	Composition	Content (g)	Dose proportion (%)	Sequence number	Composition	Content (g)	Dose proportion (%)
1	Lysine	35.35	7	27	Manganese	0.361	0.07
2	Methionine	23.57	4.668	28	Copper	2.431	0.05835
3	Phenylalanine	23.57	4.668	29	Selenium	1.5	0.035
4	Threonine	5.858	1.166	30	Chromium	0.013	0.00116
5	Tryptophan	5.858	1.166	31	Potassium	0.0093	0.00023
6	Arginine	44.21	8.753	32	Calcium	2.929	0.5835
7	Histidine	23.57	4.668	33	Magnesium	10.94	0.8753
8	Glycine	5.858	1.166	34	Inositol	36.44	1.166
9	Aspartic acid	8.838	1.75	35	Soybean phospholipid	1.7676	0.35
10	Leucine	5.858	1.166	36	Vitamin C	23.57	7
11	Isoleucine	5.919	1.166	37	Vitamin B1	0.1464	0.0292
12	Valine	8.838	1.75	38	Vitamin E	0.2947	0.05835
13	Serine	5.919	1.166	39	Glutamic acid	17.676	3.5
14	Glutamine	17.676	3.5	40	Proline	5.858	1.166
15	Taurine	4.42	0.8753	41	γ-aminobutyric acid	2.947	0.5835
16	Orotic acid	5.858	1.166	42	Egg yolk lecithin	22.69	4.085
17	Nucleotide	70.7	14	43	Cephalin	5.858	1.166
18	Vitamin A	0.0236	0.00468	44	Choline	14.645	2.9178
19	Vitamin D	0.0003	0.000058	45	α-linolenic acid	29.29	5.8356
20	Vitamin B2	0.0884	0.0175	46	γ-linolenic acid	14.645	2.9178
21	Vitamin B6	0.0884	0.0175	47	L-carnitine	8.838	1.75
22	Vitamin B12	0.0175	0.000035	48	Pentose	5.858	1.166
23	Niacin	2.929	0.5835	49	Hydroxytyrosol	29.15	1.166
24	Folic acid	0.5858	0.1166	50	Natrium carbonicum	2.653	0.5252
25	Iron	0.0262	0.0052	51	Tyrosine	5.858	1.166
26	Zinc	2.5735	0.0875	52	Cysteine	5.858	1.166

### Animal model and treatment

Male SD rats (2 months old, weighing 180 ± 26.8 g) were obtained from Animal Facility of Dalian Medical University. The animals were housed under conditions at 23 ± 2 °C with a relative humidity of 55 ± 10%. They were fed ad libitum with the standard diet and water throughout the experimental period. All of experimental procedures were approved by the Animal Studies Ethics Committee of the Dalian Medical University, China (registered number SYXK 2013-0006). A total of 75 rats were randomly assigned to five groups of 15 rats per group: Control group, D-gal group, D-gal + CE + NM, D-gal + CE, D-gal + NM. Control group subcutaneously injected with the same volume of physiological saline. The other groups were injected subcutaneously with D-gal (500 mg/kg, Sigma, St Louis, MO, USA), once daily for 90 days. Meanwhile, D-gal + CE group was treated by 1 ml embryonic chick extract for 90 days. The intake of NM was a dose-different way (1–15 days, 0.3816 g/d; 16–30 days, 0.7632 g/d; 30–90 days, 1.1448 g/d) in D-gal + NM group. D-gal + CE + NM group was received intragastric administration of CE plus NM. Rats were sacrificed at the end of treatment, and serum, organs and tissues were immediately collected for experiments.

### Histological examination

The tissue samples, such as liver, kidney, hippocampus, intestine, muscle and skin, were collected and fixed in formalin. Then the samples were dehydrated by immersion in xylene and embedded in paraffin. The tissue sections were dyed using hematoxylin and eosin (H&E) staining. Finally, the tissues from each group were examined under light microscopy.

### Determination of body weight and immune organ mass index

During the test period, rats were observed every day for signs of health, and the body weight was measured daily. The mean body weight was analyzed. The rats were weighed before humanely euthanized, the thymus and spleen were separated and weighed on an electronic tissue scale. The thymus index and spleen index were calculated. The formula was as follows: The thymus (spleen) index (mg/g) = thymus (spleen) mass (mg)/body mass (g).

### Splenocyte proliferation

Rats were killed by cervical dislocation, and spleens were aseptically removed and crushed to isolate spleen cells by grinding the spleens with a syringe plunger against nylon net. The spleen cell suspension was washed twice with RPMI-1640 (containing 10% Fetal Bovine Serum, FBS) medium and with centrifugation at 300 × g for 5 min. The recovered spleen cells were resuspended in erythrocyte lysis buffer (Solarbio, Beijing, China) for 5 min to remove erythrocytes. After centrifugation, harvested spleen cells were resuspended and then washed once in RPMI-1640-FBS medium. Spleen cells (2 × 10^6^ cell/well) were plated in triplicate in 96-well plate with ConA (10 μg/mL) or LPS (10 μg/mL). After 48 and 72 hours of culture, CCK-8 reagent (Solarbio, Beijing, China) was added, and viability was assessed by measuring the optical density (OD) at 450 nm.

### Measurement of IL-2, IL-4, IFN-γ, TGF-β

Quantitative detection of IL-2, IL-4, IFN-γ, TGF-β levels in serum was performed by using ELISA kit, according to each specific brochure (Lengton Biotech, Shanghai, China). The absorbance was measured in 450 nm wave length.

### Determination of SOD, GSH-PX, CAT, ROS, CER, MT and MDA

Liver, kidney and hippocampus were collected and homogenized with ice-cold saline. The homogenate were centrifuged at 3000 rpm 10 min, and the supernatant was used for assay. Blood serum was obtained for measure. The levels of SOD, GSH-PX, CAT, ROS, CER, MT and MDA were determined by kits (Keygen Biotech, Nanjing, China). All procedures were performed according to the manufacturer’s instructions.

### Measurement of LF content in liver and brain

A saline solution containing of brain or liver was homogenized in an ice-water. After mixing 2 ml of this homogenate with chloroform-methanol (2:1, v/v) extraction agent, the solution was centrifuged at 3000 rpm for 10 min. Then the top layer was discarded and the remaining was adjusted to 5 ml by adding chloroform-methanol mixture. Spectrofluorometric measurement was made with spectrofluorometer at an excitation maximum of 365 nm and an emission maximum of 435 nm. The fluorescence intensity of quinine bisulphate with a fresh solution (1 μg/ml) was standardized. The results were expressed as LF content of wet brain or liver.

### Morris water maze

Spatial learning and memory ability were evaluated by using the Morris water maze. Morris water maze consisted of black circular pool (150 cm in diameter, 120 cm in height, filled to a depth of 70 cm with water at 22 ± 0.5 °C). The maze was virtually divided into four equal-sized quadrants: I, II, III and IV. A circular escape platform, 8 cm in diameter, was placed in one quadrant of the pool, 2 cm below the water surface. The swimming activity was recorded using a video camera overhead and analyzed via a computer image analyzer system. In the acquisition training session, each rat was given four trials consecutively per day. In each trial, the time a rat spent from being put into the water to finding and climbing onto the hidden platform was recorded as escape latency. The rats that found the platform was allowed to stay on it for 10 s and then taken back to its cage for 40 s inter-trial interval. If the rats did not locate the escape platform within 90 s, it was placed on the platform and stayed there for 10 s, and its escape latency was recorded as 90 s. On the sixth day, the rats were released from the quadrant opposite from the previous platform and allowed to swim freely for 120 s. The total number of each rat crossed the position where the escape platform was once placed (crossing number) and time it spent in the target quadrant (where the platform was once hidden) was recorded.

### Measurement of glutamate

After Morris water maze test, the brain tissues were immediately dissected out and rinsed with PBS to remove excess blood. The brain tissues were homogenized in cold PBS. After centrifugation at 3000 × g at 4 °C for 10 min, the supernatants were collected and waited for analysis. The level of glutamate was determined according to the assay kit providers’ instructions (Jiancheng Biotech, Nanjing, China).

### Western blot

Splenic B lymphocytes were isolated and treated with LPS (concentration 1 µg/ml, Sigma, St Louis, MO, USA) for 24 h. Proteins were electrophoresed by 10% SDS-PAGE. The separated proteins were transferred to a polyvinylidene difluoride membrane. After blocking for 2 h with phosphate-buffered saline containing 0.1% Tween 20 and 5% powdered skim milk, the membrane was incubated with anti-NF-κB p-65 antibody and anti-TLR4 antibody (1:1000 Abcam, Cambridge, UK), anti-GAPDH antibody (1:2000 Bioworld, Minnesota, USA) overnight in 5% powdered skim milk buffer. Then the blots were washed and incubated with secondary antibodies. All bands were detected using the enhanced chemiluminescence (Amersham Biosciences, Buckinghamshire, UK) and analyzed by LabWorks (TM ver4.6,UVP, BioImaging systems).

### Statistical analysis

All data were analyzed using SPSS 17.0 statistical packages (SPSS Inc., Chicago, IL). Data were expressed as means ± standard deviation (SD). Each experiment was performed at least in triplicate, and the measurements were performed in three independent experiments. Paired t-test was applied to compare data from two groups. Two-way ANOVA was used for multiple comparisons between pairs. A value of P < 0.05 was considered to be statistically significant.

### Animal statement

Animal experiments were performed following the approved international guide for the Care and Use of Laboratory Animals, including any relevant details. All experiments were approved by the Committee on the Ethics of Animal Experiments of the Dalian Medical University, China.

## Supplementary information

Dataset 1

## Data Availability

The datasets generated and analyzed during the current study are available from the corresponding author on reasonable request.
