# Author Correction: Combination of chick embryo and nutrient mixture prevent D-galactose-induced cognitive deficits, immune impairment and oxidative stress in aging rat model

**DOI:** 10.1038/s41598-020-61999-9

**Published:** 2020-03-13

**Authors:** Jia Ma, Huaxin Wang, Bing Liu, Yujia Shan, Huimin Zhou, Xia Qi, Wenguo Wu, Li Jia

**Affiliations:** 1 College of Laboratory Medicine, Dalian Medical University, Dalian, 116044 Liaoning Province China; 2 Department of Pathology and Forensic Medicine, Dalian Medical University, Dalian, 116044 Liaoning Province China; 3 Dalian Jinfu Biological Technology Development Co., Ltd, Dalian, 116000 Liaoning Province China

Correction to: *Scientific Reports* 10.1038/s41598-019-40953-4, published online 11 March 2019

This Article contains errors, whereby Figure 2 was misassembled. As a result, the following panels are incorrect:

The kidney and hippocampus images in the Control group

The liver, kidney and hippocampus images in the D-gal group

The hippocampus and skin images in the D-gal+CE+NM group

The liver and kidney images in the D-gal+CE group

The kidney and skin images in the D-gal+NM group

The correct Figure 2 appears below as Figure 1.

**Figure 1:**
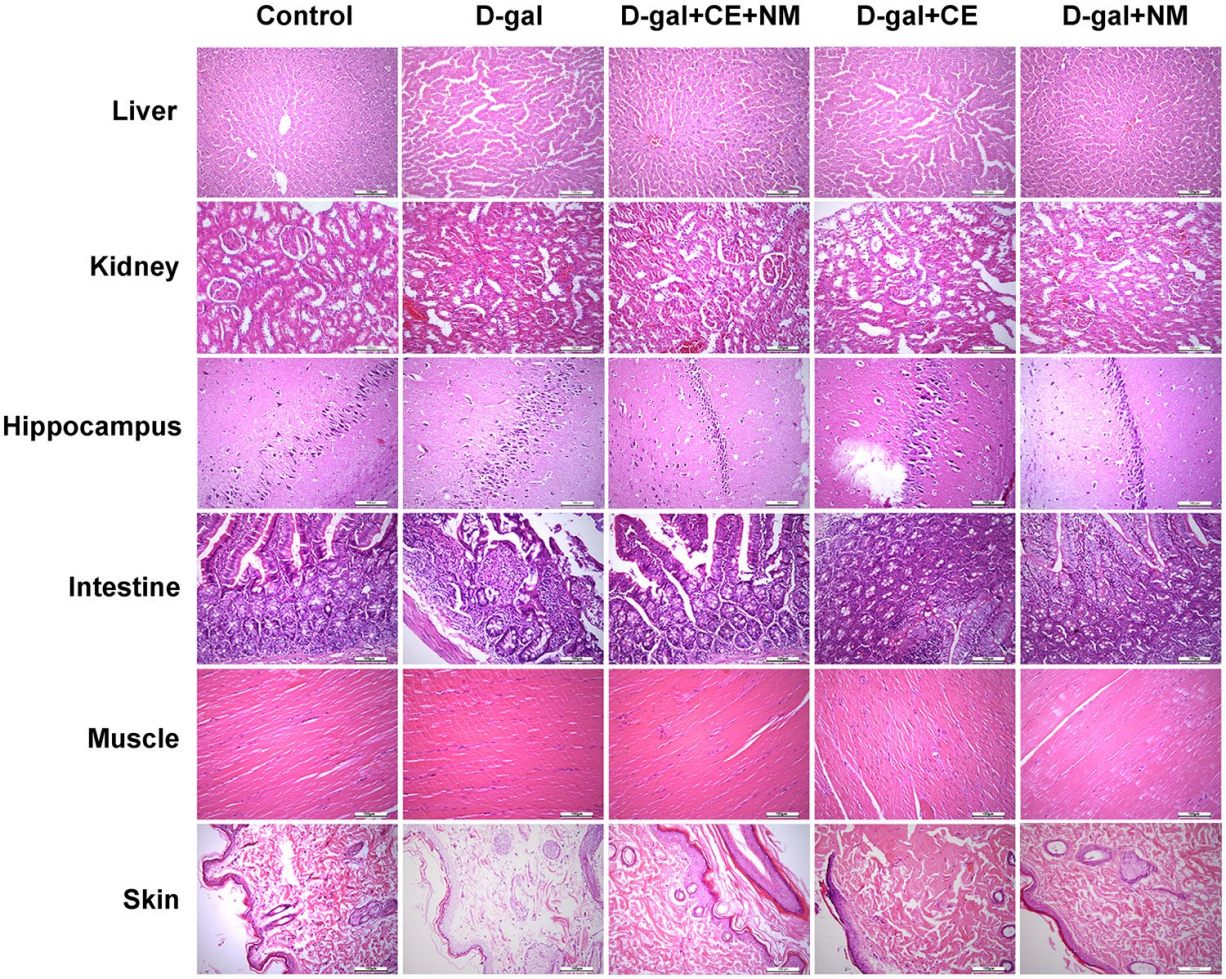
.

These changes do not affect the conclusions of the Article.

