# Supplementary material for: Combination of chick embryo and nutrient mixture prevent D-galactose-induced cognitive deficits, immune impairment and oxidative stress in aging rat model
Source: Sci Rep. 2019 Mar 11;9:4092. doi: 10.1038/s41598-019-40953-4 (PMC6411858; doi:10.1038/s41598-019-40953-4)
Supplement: Supplementary file 1 — Dataset 1 [file 41598_2019_40953_MOESM1_ESM.pdf]

Supplementary Information for

Combination of chick embryo and nutrient mixture prevent D-galactose-induced cognitive deficits, immune impairment and oxidative stress in aging rat model

Jia Ma, Huaxin Wang, Bing Liu, Yujia Shan, Huimin Zhou, Xia Qi, Wenguo Wu, Li Jia,*

*Correspondence and requests for materials should be addressed to L.J. (jiali0386@sina.com)

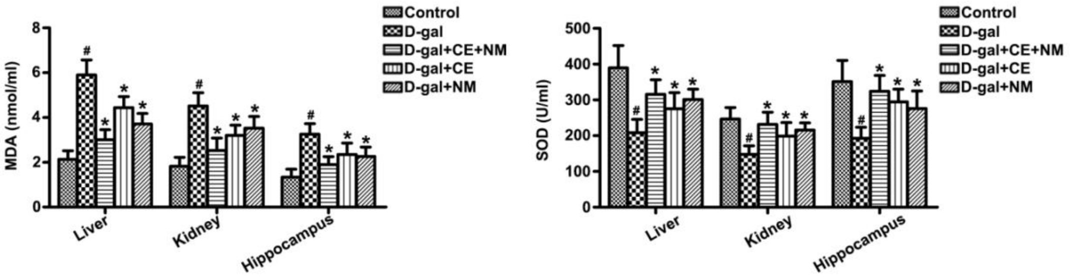

Fig.S1. CE and NM supplement effect on MDA level and SOD activity of liver, kidney and hippocampus tissues in aging rats. #P<0.05 versus control groups. *P<0.05 versus D-gal groups. Data are expressed as mean ± SD of three independent experiments.

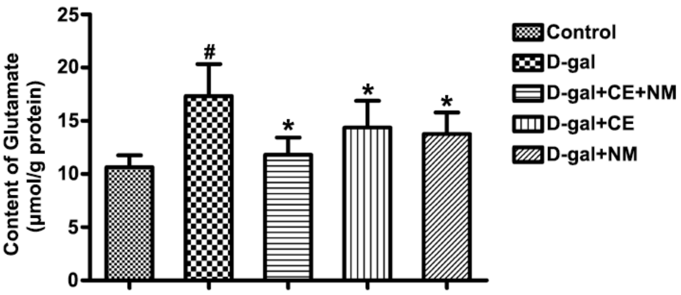

Fig.S2. CE and NM supplement effect on glutamate content of brain tissues. #P<0.05 versus control groups. *P<0.05 versus D-gal groups. Data are presented as mean ± SD

of three independent experiments.

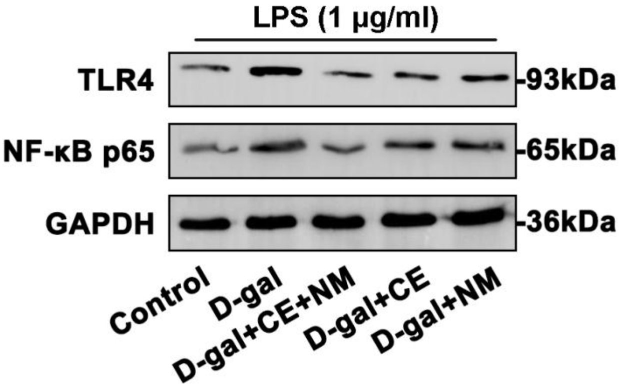

Fig.S3. CE and NM supplement effect on TLR4/NF-κB pathway induced by LPS in splenic B lymphocytes for 24 h. The protein levels of TLR4 and NF-κB p65 in splenic B lymphocytes were determined by western blot (The samples derive from the same experiment and that blots were processed in parallel).
